# Analysis of Fractal Correlation Properties of Heart Rate Variability during an Initial Session of Eccentric Cycling

**DOI:** 10.3390/ijerph181910426

**Published:** 2021-10-03

**Authors:** Bruce Rogers, Thomas Gronwald, Laurent Mourot

**Affiliations:** 1 College of Medicine, University of Central Florida, 6850 Lake Nona Boulevard, Orlando, FL 32827-7408, USA; 2 Faculty of Health Sciences, Department of Performance, Neuroscience, Therapy and Health, MSH Medical School Hamburg, University of Applied Sciences and Medical University, Am Kaiserkai 1, 20457 Hamburg, Germany; Thomas.Gronwald@medicalschool-hamburg.de; 3 EA3920 Prognostic Factors and Regulatory Factors of Cardiac and Vascular Pathologies, Exercise Performance Health Innovation (EPHI) Platform, University of Bourgogne Franche-Comté, 25000 Besançon, France; laurent.mourot@univ-fcomte.fr; 4 Division for Physical Education, Tomsk Polytechnic University, 634040 Tomsk, Russia

**Keywords:** eccentric cycling, HRV, detrended fluctuation analysis, DFA a1, endurance exercise

## Abstract

Eccentric cycling (ECC) has attracted attention as a method to improve muscle strength and aerobic fitness in populations unable to tolerate conventional methods. However, agreement on exercise prescription targets have been problematic. The current report is an initial exploration of a potentially useful tool, a nonlinear heart rate (HR) variability (HRV) index based on the short-term scaling exponent alpha1 of detrended fluctuation analysis (DFA a1), which has been previously shown to correspond to exercise intensity. Eleven male volunteers performed 45 min of concentric (CON) cycling and ECC separated by 1 month. Work rates were matched for HR (~50% of the maximal HR) during the first 5 min and remained stable thereafter. HRV, HR, oxygen consumption (VO_2_), and cycling power were monitored and evaluated at elapsed times of 10 (T10) and 45 (T45) minutes duration. HR significantly increased between ECC T10 and ECC T45 (*p* = 0.003, d = 1.485), while DFA a1 significantly decreased (*p* = 0.004, d = 1.087). During CON, HR significantly increased (*p* < 0.001 d = 1.570) without significant DFA a1 change (*p* = 0.48, d = 0.22). Significantly higher HR was observed at T45 in ECC than in CON (*p* = 0.047, d = 1.059). A session of unaccustomed ECC lead to decreased values of DFA a1 at T45 in comparison to that seen with CON at similar VO_2_. ECC lead to altered autonomic nervous system balance as reflected by the loss of correlation properties compared to CON.

## 1. Introduction

Eccentric cycling (ECC) has garnered interest as an exercise modality to promote locomotor muscle improvements at reduced cardiovascular demands compared to conventional concentric cycling (CON) [1,2,3]. ECC refers to the muscular action of actively opposing the motion of motor driven bicycle pedals moving in the reverse direction [1]. Although it may appear that the participant is pedaling in reverse, they are actually attempting to stop the pedals moving backward, thereby engaging locomotor muscles in an eccentric fashion. At an equivalent metabolic cost or oxygen consumption (VO_2_), ECC can be performed at much higher power levels, resulting in enhancement of muscular size, strength, and oxidative properties [1,4,5,6]. However, it appears that ECC cycling offers no benefit for peak aerobic training over CON methods [5,7]. Therefore, this modality is particularly suited for rehabilitation purposes in patients with cardiovascular or pulmonary disease who are unable to mount sufficient muscular pedaling force due to inherent limitations. Unfortunately, recommendations for ECC training intensity are problematic. At the same VO_2_, ECC may lead to a higher heart rate (HR), making suggestions based on common HR based formulas unreliable [1,5,8]. Many of the ideal candidates for an ECC program may be taking medications that can affect HR, such as beta-adrenergic blocking agents, further obscuring HR related intensity target formulas. In addition, the power to VO_2_ relationship may not be equivalent to that done with CON since the oxygen cost per watt of each modality is different [1,5]. Although cardiovascular testing centers may be able to evaluate individuals during CON, gas exchange testing with ECC equipment may not be available. Therefore, alternate means to evaluate exercise intensity during aerobic eccentric exercise that do not rely on HR or power and do not require prior incremental ramp testing with gas exchange would be helpful.

Changes in heart rate variability (HRV) indexes during dynamic aerobic exercise have been well studied over the past several decades [9]. The concept revolves around the characterization of the variation of the cardiac beat to beat sequence over time. Although several linear, time, and frequency domain measures, such as SDNN (standard deviation of normal-to-normal RR intervals), high frequency (HF) power, as well as nonlinear measures of HRV such as SD1 (standard deviation 1 from Poincaré plot analysis), have been shown to have potential to assess exercise intensity, all reach a nadir near the aerobic threshold, limiting their ability to estimate loads above this point [10]. In addition, they require a dedicated exercise ramp to failure for standardization to HR, VO_2_, or power. Recently, a nonlinear HRV index, the short-term exponent alpha 1 of detrended fluctuation analysis (DFA a1) based on fractal correlation properties, has been shown to decline with increasing exercise loads throughout all intensity domains providing information about physiological transitions corresponding to the aerobic and anaerobic threshold (e.g., as represented by the first and second ventilatory thresholds) [10,11,12]. Fractal behavior in relation to HRV can be described as degrees of self-similarity between RR interval variations over different time scales [13]. At low exercise intensity, DFA a1 values are usually in a well correlated fractal range near or above 1.0. As exercise intensity rises, DFA a1 passes 0.75 at the aerobic threshold and continues to drop to an uncorrelated random pattern (0.5) near the anaerobic threshold, finally reaching below 0.5, signifying an anticorrelated range at the very highest work rates [10,11,12]. The anticorrelated state can be viewed as a protective feedback and stabilizing mechanism, where interactions and/or coordination of subsystems fail before the entire system fails [13,14]. Another unique advantage of DFA a1 for intensity monitoring revolves around its dimensionless nature, which makes standardization to other internal load parameters such as gas exchange or lactate unnecessary. For example, an intensity associated with a DFA a1 value of 0.75 corresponds to a workload near the aerobic threshold in a wide spectrum of individuals and is referred to as the HRVT (heart rate variability threshold) [11,15].

As exercise intensity rises, there is a withdrawal of parasympathetic and enhancement of sympathetic influence that leads to a decline in DFA a1 [10,16]. On the other hand, it has been shown that, for the same VO_2_ or HR, ECC is associated with an increased muscle tension and sympathetic stress [1,8], especially when participants are not accustomed to eccentric exercise, and ECC should produce the greatest degree of muscle soreness. However, to the best of our knowledge, it is not known whether this is accompanied by a more pronounced decrease in DFA a1. DFA a1 behavior could offer an opportunity to monitor exercise loads and interaction patterns from the autonomic system standpoint during ECC. Therefore, the aim of this report is to provide an initial observation of DFA a1 activity during ECC compared to conventional, CON. DFA a1 will be evaluated at the beginning and end of a representative 45 min exercise session consisting of either low-intensity ECC or CON. We anticipate that DFA a1 will show a higher decrease during ECC compared to CON.

## 2. Methods

### 2.1. Participants

Eleven male volunteers who had no history of cardiovascular disease, prescribed medication, smoking, and were already training with endurance related exercise several times per week were evaluated. None had performed eccentric related activity, including prolonged downhill running. The mean participant age was 28 ± 6 years, and mean body mass index (BMI) was 23 ± 3 kg/m^2^. All procedures were in accordance with the ethical standards of the institutional research committee (RCB number 2014-A00501-46) and conformed to the principles of the Declaration of Helsinki, and all subjects gave their informed consent for inclusion before they participated in the study.

### 2.2. Baseline Assessment

Baseline gas exchange testing was performed using an incremental maximal cycling exercise test performed on a braked ergometer (Excalibur Sport, Lode, Groningen, Netherlands). After a stable resting period, a 3 min warm-up was performed at 50 watts, and the intensity was increased by 25 watts every minute until volitional exhaustion. The HR (CASE P2, GE Healthcare, Buckinghamshire, UK) at the first ventilatory threshold (VT1) was determined using pulmonary gas exchange data (MGC Diagnostics—Ultima CPXTM System, St Paul, Minnesota, USA) and the ventilatory equivalents method [17]. Maximal oxygen uptake (VO_2MAX_) was defined as reaching a VO_2_ plateau or a HR > 90% of predicted maximal HR (220—age), and the corresponding maximal heart rate (HR_MAX_) and power output were reported.

### 2.3. Eccentric and Concentric Cycling Exercise Sessions

After the baseline cycling exercise test, a low-intensity 5 min familiarization session was performed by participants to learn eccentric cycle ergometer coordination. The eccentric cycle used was a semi-recumbent prototype (developed by Inserm U887 and Tech med, Champs sur Yonne, France), which permits different pedaling frequencies. The ergometer was pedaled backward with participants actively resisting the movement. A visual display of cycling torque produced during ECC was provided. In order to maintain comparable hemodynamics during CON, a semi-recumbent concentric ergometer was used (Excite + Recline, Technogym SpA, Cesean, Italy). ECC and CON exercise sessions were randomized, counterbalanced, and were performed one month apart to prevent any delayed onset muscle soreness and the long-term protective, repeat bout effect triggered by eccentric cycling [18]. All cycling sessions were performed at 30 rpm for 45 min. This cadence was chosen because performing high velocity eccentric contractions requires an advanced degree of coordination that needs several training sessions not performed in this study, which focused on an acute bout of exercise. Moreover, the custom-made ergometer presented technical limitations and could not allow the achievement of high pedaling frequencies. ECC and CON cycling power were selected to produce a similar HR of 100 bpm at the beginning of exercise (first 5 min) and remained stable throughout the exercise period. The target HR was fixed based on a previous study, which was around 54% of peak heart rate observed during a concentric incremental test [19]. To confirm that the work was practical for the participants, a 5 min test interval was performed on the ECC cycle ergometer immediately before the ECC bout. Since the initial session of eccentric cycling should produce the greatest degree of muscle soreness and potential change in autonomic balance [18], testing was performed without adaptation to eccentric activity. VO_2_ was continuously monitored by gas exchange testing during all sessions (Vmax Encore 29 C, SensorMedics, Yorba Linda, CA, USA).

### 2.4. RR Measurements and Calculation of DFA a1

An ECG device with a sampling rate of 500 Hz (CASE P2, GE Healthcare, Buckinghamshire, UK) was used to detect and record RR intervals during all sessions. RR data was downloaded as text files, then imported into Kubios HRV Software Version 3.5 (Biosignal Analysis and Medical Imaging Group, Department of Physics, University of Kuopio, Kuopio, Finland; [20]). Kubios preprocessing settings were set to the default values including the RR detrending method which was kept at “Smoothn priors” (Lambda = 500). The RR series was then corrected by the Kubios “automatic method” [21] and relevant parameters exported as text files for further analysis. In brief, DFA a1 estimation is based on the root mean square of the fluctuation of the detrended RR data and is measured in observation windows of different sizes (widths). The data is then plotted against the size of the window on a log–log scale. The scaling exponent represents the slope of the line, which relates (log) fluctuation to (log) window size with DFA a1 window width set to 4 ≤ *n* ≤ 16 beats for computation of the a1 component [10,13].

DFA a1 and average HR were calculated from the RR data series of the 2 min time window at the end of 10 (T10) and 45 min (T45) of cycling. Two-minute time windowing was selected based on previous estimates as to the minimal beat count required [22]. Artifact levels measured by Kubios HRV were below 5%. This limit was previously shown to have minimal effect on DFA a1 during exercise [23].

### 2.5. Blood Pressure and Biochemical Measurements

Continuous monitoring of diastolic and systolic blood pressure was obtained via a photoplethysmographic device using the model flow method (Nexfin, BMEYE B.V., Amsterdam, The Netherlands). At the end of exercise, whole blood lactate concentrations were measured from venous samples collected into lithium heparin coated vacutainers (BD Vacutainer, Fisher Scientific, Strasbourg, France) using a portable lactate analyzer (Lactate Pro, Arkray, Kyoto, Japan). Additionally, separate samples were collected at the same time point in ethylenediaminetetraacetic acid (EDTA) to determine norepinephrine concentration using a specific and sensitive radioenzymatic method at the Clinical Chemistry Unit at University Hospital of Besançon.

### 2.6. Statistics

Statistical analysis of means and standard deviations were performed for demographic and performance data in Excel 365 (Microsoft, USA). The Shapiro–Wilk test was used to confirm normal distribution of the data. Levene’s test was used to show that the between group difference in variances was equal. The correlation between the percent difference in HR, systolic, diastolic blood pressure, norepinephrine, and serum lactate levels with the percent difference in DFA a1 at T45 of ECC vs. CON session ((ECC T45—CON T45/CON T45) × 100) was assessed using linear regression, Pearson’s r correlation coefficient, and standard error of estimate (SEE). The strength of Pearson’s r correlations evaluated as follows: 0.3 ≤ r < 0.5 low; 0.6 ≤ r < 0.8 moderate; and r ≥ 0.8 high [24]. Two-way ANOVA with repeated measures and Bonferroni correction was used for main effect analysis (time: T10 vs. T45, group: CON vs. ECC) and paired t testing for direct comparisons. For all tests, the statistical significance was accepted as *p* ≤ 0.05. Cohen’s d was used to denote effect sizes for direct comparisons (small effect = 0.2, medium effect = 0.5, large effect = 0.8; [25]. ANOVA statistical analysis was performed using SPSS 23.0 (IBM Statistics, USA).

## 3. Results

**Figure 1:**
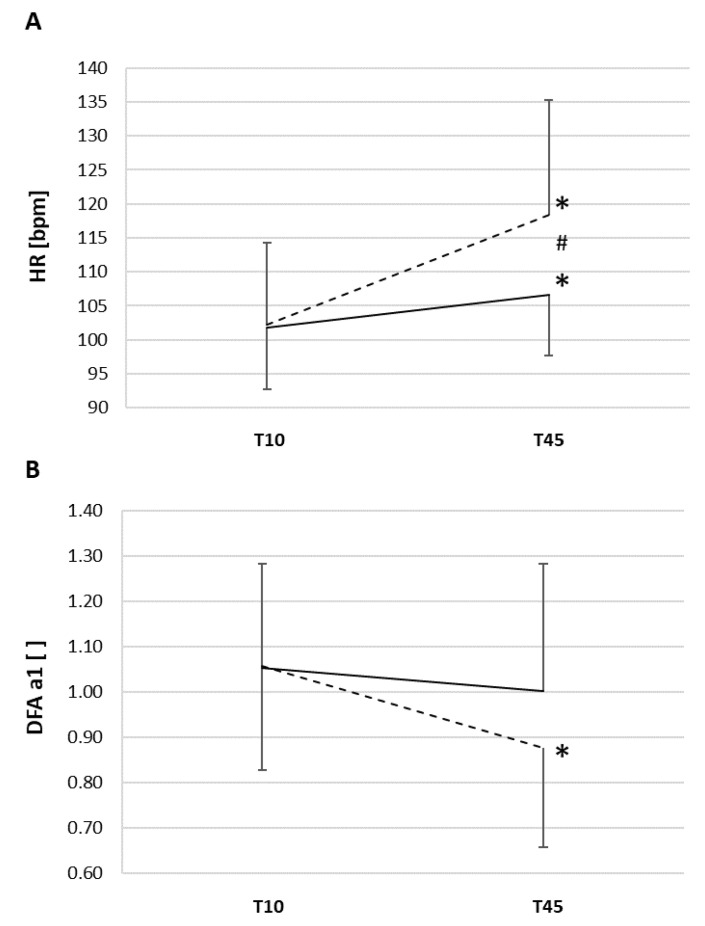
A: HR during ECC and CON exercise session at elapsed time 10 (T10) and 45 min (T45). B: DFA a1 during ECC and CON exercise session at elapsed time 10 (T10) and 45 min (T45). ECC: dashed line, CON: solid line, error bars represent standard deviation, * significant for direct comparison of T10 vs. T45, ^#^ significant for direct comparison of CON vs. ECC at T10 or T45.

Incremental cycling test results showed a mean VO_2MAX_ of 51 ± 10 mL/kg/min, HR_MAX_ of 189 ± 5 bpm, maximal power of 318 ± 69 watts, and heart rate at first ventilatory threshold of 153 ± 6 bpm (81% of HR_MAX_). Individual results of the study are listed in Table 1 for HR, VO_2_, and DFA a1 for both CON and ECC at T10 and T45 of the exercise sessions. Figure 1 shows HR and DFA a1 during ECC and CON at T10 and T45. As expected, HR at T10 were 102 ± 9 bpm and 102 ± 13 bpm for CON and ECC, respectively (corresponding to 54% of HR_MAX_), without significant difference. The corresponding cycling power was 81 ± 15 watts for CON and 205 ± 31 watts for ECC (*p* < 0.001, d = 4.415). Significant interaction effects of time and group could be found for HR (F = 7.084, *p* = 0.015, partial η^2^ = 0.262). While the HR differential was not significant at T10, a progressive drift over time was observed (F = 25.053, *p* < 0.001, partial η^2^ = 0.556), and at T45, the HR were significantly higher for both CON (*p* < 0.001, d = 1.570) and ECC (*p* = 0.003, d = 1.485), with significantly higher HR for ECC than CON at T45 (*p* = 0.047, d = 1.059). Contrary to HR, VO_2_ showed no significant changes in both conditions (F = 0.930, *p* = 0.346, partial η^2^ = 0.044). Finally, the ANOVA analysis showed significant main time effects for DFA a1 (F = 7.432, *p* = 0.013, partial η^2^ = 0.271). DFA a1 did not significantly change during CON (*p* = 0.48, d = 0.22 but significantly decreased during ECC with large effect size (*p* = 0.004, d = 1.087). Values at T45 for norepinephrine, lactate, systolic, and diastolic blood pressure for each participant are listed in Table 2. No significant differences were observed between values at the end of CON and ECC for norepinephrine (*p* = 0.28, d = 0.35), lactate (*p* = 0.71, d = 0.12), and systolic blood pressure (*p* = 0.43, d = 0.25). However, diastolic blood pressure was significantly higher at the end of ECC compared to CON (*p* = 0.01, d = 0.91).

**Figure 2:**
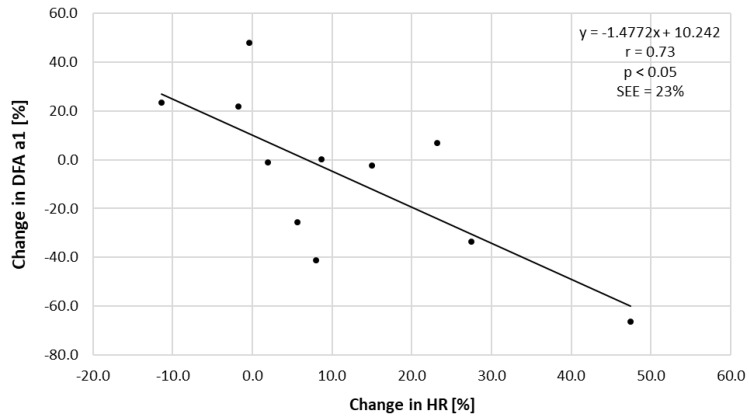
Correlation between the percent difference in HR and DFA a1 at T45 of ECC vs. CON session ((ECC T45—CON T45/CON T45) × 100). Pearson’s r, *p* value and SEE (standard error of estimate) listed.

The percentage difference of DFA a1 at T45 of ECC vs. CON sessions did correlate with the percent difference in HR with Pearson’s r of 0.73 (see Figure 2). The percentage difference of DFA a1 did not correlate with that of norepinephrine (r = 0.31, SEE = 32%, *p* = 0.35), serum lactate (r = 0.07, SEE = 33%, *p* = 0.32), systolic (r = 0.18, SEE = 33%, *p* = 0.59) and diastolic blood pressure differences (r = 0.07, SEE = 32%, *p* = 0.83).

## 4. Discussion

The intent of this study was to explore the behavior of the nonlinear HRV index DFA a1 during eccentric cycling. In view of the potential usage of this type of exercise for cardiac disease rehabilitation [26], a clinically relevant question is whether an unaccustomed bout of moderate duration ECC may lead to a change in DFA a1 compared to CON. Since ECC may trigger higher sympathetic activity than CON [1,8], along with complex interaction between mechanoreceptors, metaboreceptors and baroreceptors, a greater decline in DFA a1 was anticipated. In this study, the initial workload was set to have the same HR at the beginning of CON and ECC (54% of HR_MAX_). As expected, the power output was higher during ECC than CON and remained stable throughout the 45 min of exercise. VO_2_ remained stable and similar in both CON and ECC at T10 and T45. However, the results indicate a heterogenous response in HR and DFA a1 at ECC T45, despite a cycling intensity well below the first ventilatory threshold. In regard to mean HR, the results were consistent with what has been reported with a significant rise in ECC T45 values [1,8]. DFA a1 showed a significant decrease in T45 during ECC on a group level, with some participants having deep declines. When assessed at 10 min elapsed time, no significant difference in either DFA a1 or HR were seen between CON or ECC. Of interest, the difference in DFA a1 between ECC vs. CON was correlated with that of the HR (r = 0.73), meaning that the more the HR increased during ECC vs. CON, the more DFA a1 decreased. In contrast, the decline in DFA a1 was not well correlated with the differences seen at T45 for norepinephrine, lactate, or systolic or diastolic blood pressure. Further studies are required to better understand the mechanisms behind the DFA a1 decrease with ECC exercise. However, the implication of the particular DFA a1 values from the exercise intensity and “organismic demand” [10] standpoints should be noted. Mean DFA a1 during both ECC and CON at the 10 min mark, as well as CON at 45 min, were about 1.0, corresponding to well correlated fractal patterns normally seen at very low exercise intensities well below the VT1 [10,11]. In contrast, mean DFA a1 during the ECC T45 sampling interval was 0.877, with 5 out of 11 participants having values near or below the 0.75 value. A DFA a1 of 0.75 has been associated with intensities near the VT1 during incremental tests while cycling with conventional, concentric contractions or running [11,15]. One participant had a value below 0.5, which corresponds to exercise intensities above the anaerobic threshold [12]. This same participant showed the largest HR rise during ECC T45 of nearly 50 bpm. The design of the present study does not allow us to fully explain such behavior. However, we believe that DFA a1 monitoring in patients with cardiovascular disease may help to forewarn inappropriate exercise intensities during ECC during rehabilitation training.

One of the major attractions of ECC, especially in cardiac rehabilitation scenarios, revolves around the concept of training with more challenging muscular loads at a relatively low oxygen consumption [1,2,3,4,5]. This is felt to encourage muscular growth along with oxidative metabolic shifts despite a reduced cardiac strain during training. However, recommendations for training intensity distributions have been problematic due to the altered HR and/or power relationships to oxygen consumption during eccentric exercise [1]. In addition, many of the best candidates for an ECC program, such as those with congestive heart failure or ischemic disease will be on beta adrenergic blockade, further altering heart rate targets [27]. Even under optimal circumstances, prescribing training intensity targets based on predetermined formulas can be problematic [28,29]. Leveraging the well-established relationship of DFA a1 to exercise intensity levels [10,11,12] may therefore provide needed exercise prescription guidance. This could be in the form of estimating an ECC related aerobic threshold, as well as prevention of excessive exercise intensities. The present results also point to the possibility of untoward autonomic stress, as manifested by a decline in DFA a1 during the initial acclimation phases of ECC in some participants. Previous studies have noted a relative rise in HR during ECC compared to CON at the same VO_2_ [1,8]. This has been hypothesized to be due to a primary reduction in stroke volume with a secondary rise in HR [8]. Underlying factors involved include higher vascular afterload, altered respiratory kinetics, rib cage expansion, and noradrenaline increase [1,8]. However, it is also possible that the rise in HR is the primary event related to a change in autonomic balance. The autonomic driven, primary rise in HR would therefore lead to the stroke volume reduction. This scenario is perhaps analogous to that of HR elevation during exercise in the heat [30]. Over the years, it has been argued whether the HR rise during heat exposure is due to cutaneous volume sequestration with a resulting decline in preload/stroke volume (and secondary HR elevation) vs. heat exposure induced autonomic alteration with a primary HR rise (and secondary stroke volume reduction). In a series of studies [30], it has been shown that the later scenario (primary HR elevation) may be the underlying mechanism, illustrating the importance of autonomic balance in exercise physiology. In fact, skin and core body temperature have been shown to correlate with the degree of HR elevation seen during ECC [31]. It would be of interest to see whether body temperature elevation also relates to the heterogeneous DFA a1 decline in this situation, as an influencing factor due to organismic system demands. The rise in core temperature during ECC may provide the common linkage to the change in autonomic balance seen.

## 5. Limitations and Future Directions

The present study investigated the effects of an initial bout of unaccustomed ECC on DFA a1 over time compared to CON. As has been previously shown, slow acclimation to ECC training is important to minimize discomfort, local muscular damage, and inflammatory changes [18]. It is certainly possible that gradual acclimation training would have produced different results to what was seen here but might not have provided the stimulus we wanted to provoke for this initial study. No female participants were included, and further study comparing male and female response is recommended. An interesting metric that was not assessed in our study was skin and core body temperature. Given the relationship of core temperature and HR elevation during ECC [30], future studies examining DFA a1 could incorporate this as part of the monitoring protocol. RR data were acquired with an ECG, not a typical consumer heart rate monitoring device. Recent study indicates minimal device bias between high quality ECG vs. consumer wearables [22]; therefore, an extension of DFA a1 agreement to commonly used chest belt monitoring devices seems reasonable. A significant limitation to the use of DFA a1 for exercise intensity assessment in general is the presence of artifact and subsequent correction. Artifact levels in this study were below 5%, which has been shown to have minimal effect on DFA a1, but RR data with greater degrees of artifact may be unreliable [23]. Recent work points to specific DFA a1 values that correspond to either the aerobic (0.75) or anaerobic (0.5) threshold in recreational runners [11,12]. In addition, the 0.75 transition concept appears to be valid in a population with heart failure, ischemic heart disease, and beta blocker usage [15]. If the variable DFA a1 decline seen with unaccustomed ECC is due to a corresponding variation in autonomic stress, real-time monitoring of this metric could be of great value in assuring patient safety as well as providing individual training intensity prescription guidelines. A smartphone application developed for many available devices (Android and iOS) using consumer grade heart rate monitors has also been explored for both DFA a1 threshold testing and in adhering to a polarized training model [32]. Further assessment of the usage of DFA a1 for real-time monitoring during ECC seems a worthwhile endeavor especially in at-risk patient populations.

## 6. Conclusions

During a session of unaccustomed ECC, DFA a1 and HR were stable at an elapsed time of 10 min but had variable changes at 45 min in comparison to CON at similar oxygen consumption below the first ventilatory threshold. Although mean HR rose and DFA a1 declined after 45 min of ECC, some participants had variable responses. The rise in HR was correlated with the decline in DFA a1, pointing toward changes in autonomic balance as a potential mechanism. Given the interest in ECC as an initial rehabilitation modality for those with cardiovascular disease, further research into DFA a1 behavior during this activity for training intensity guidance is recommended.

## Figures and Tables

**Table 1:** Heart rate (HR), oxygen consumption (VO_2_), and DFA a1 for all participants measured at elapsed time of 10 (T10) and 45 (T45) minutes of either concentric (CON) or eccentric (ECC) cycling. Mean (± standard deviation, SD) listed. * Significantly different from T10; ^#^ significantly different from CON.

	T10						T45					
Participant	HR (bpm)		VO_2_ (mL/min/kg)		DFA a1		HR (bpm)		VO_2_ (mL/min/kg)		DFA a1	
	CON	ECC	CON	ECC	CON	ECC	CON	ECC	CON	ECC	CON	ECC
1	105	104	17.6	14.3	0.946	1.18	105	93	18.3	14.0	0.854	1.053
2	93	88	18.7	15.1	1.381	1.323	97	103	18.6	12.9	1.595	1.187
3	101	120	19.5	35.9	1.537	0.722	102	150	21.7	20.4	1.202	0.404
4	107	103	15.4	17.9	0.926	1.272	115	146	14.4	19.4	1.109	0.736
5	83	75	17.3	12.8	1.155	1.004	90	98	17.9	15.9	1.339	0.79
6	97	115	14.9	20.0	0.761	1.083	101	125	14.9	19.3	0.903	0.965
7	100	102	15.5	13.5	0.973	1.403	110	119	17.2	17.6	1.076	1.077
8	97	107	20.0	19.8	1.004	0.707	100	115	20.1	15.4	0.682	0.666
9	105	90	18.4	15.1	0.856	0.81	110	108	19.7	14.5	0.706	0.86
10	118	113	16.8	13.4	1.188	0.971	120	123	15.4	15.6	0.804	0.795
11	114	108	13.9	9.6	0.851	1.161	122	121	15.7	10.7	0.756	1.118
Mean (±SD)	102 (±9)	102 (±13)	17.1 (±1.9)	17.0 (±6.7)	1.053 (±0.228)	1.058 (±0.228)	107 (±9) *	118 (±17) *^#^	17.6 (±2.2)	16.0 (±2.9)	1.002 (±0.277)	0.877 (±0.221) *

**Table 2:** Norepinephrine, serum lactate, and systolic and diastolic blood pressure (BP) for all participants measured at elapsed time of 45 min of either concentric (CON) or eccentric (ECC) cycling. Mean (± standard deviation, SD) listed. ^#^ significantly different from CON.

Participant	Norepinephrine (pg/mL)		Serum Lactate (mmol/L)		Systolic BP (mmHg)		Diastolic BP (mmHg)	
	CON	ECC	CON	ECC	CON	ECC	CON	ECC
1	715	513	1.79	1.33	127	141	57	76
2	696	783	1.72	2.36	128	143	61	71
3	580	753	1.55	1.89	135	115	56	62
4	820	1355	2.10	2.22	121	149	61	85
5	363	317	1.73	1.45	144	135	70	65
6	476	810	1.46	2.06	186	158	82	78
7	359	489	1.53	1.93	148	153	68	81
8	482	475	1.28	1.20	118	148	58	80
9	449	365	2.18	1.41	147	148	85	84
10	568	446	1.64	1.70	129	136	69	74
11	423	440	1.91	1.90	140	147	73	86
Mean (±SD)	539 (±144)	613 (±284)	1.72 (±0.26)	1.77 (±0.36)	138 (±18)	143 (±11)	67 (±9)	77 (±7) ^#^

## Data Availability

The raw data supporting the conclusions of this article will be made available by the authors, without undue reservation.
